# Anxiety, Self‐Efficacy, and Their Correlates Among Caregivers of Patients With Ostomy: A Cross‐Sectional Study in Southeastern Iran During 2023–2024

**DOI:** 10.1002/hsr2.72517

**Published:** 2026-05-13

**Authors:** Sima Mokhtarabadi, Ala Shamsi, Haadiyeh Rahbari, Marzie Mosa Ali, Mohamed Alnaiem, Mahlagha Dehghan

**Affiliations:** ^1^ Nursing Research Center Kerman University of Medical Sciences Kerman Iran; ^2^ Nursing Research Center Kerman Medical Sienese University Kerman Iran; ^3^ Bahonar Hospital Kerman University of Medical Sciences Kerman Iran; ^4^ Mental Health Services, Hamad Medical Corporation Doha Qatar; ^5^ HIV/STI Surveillance Research Center, and WHO Collaborating Center for HIV Surveillance, Institute for Futures Studies in Health Kerman University of Medical Sciences Kerman Iran; ^6^ Reproductive and Family Health Research Center Kerman University of Medical Sciences Kerman Iran

**Keywords:** anxiety, caregiver, patients with ostomy, self‐efficacy, Southeastern Iran

## Abstract

**Background and Aims:**

Caregivers of patients with ostomy often face physical and psychological challenges, including anxiety. Their ability to provide specialized care is closely linked to their sense of self‐efficacy. This study aimed to assess anxiety and self‐efficacy among caregivers of patients with ostomy.

**Methods:**

This cross‐sectional study was conducted at a stoma clinic in Kerman province, Iran, from May 2, 2023 to July 20, 2024. Data were collected from 120 participants using a demographic questionnaire, the Hamilton Anxiety Rating Scale (HAM‐A), and the Generalized Self‐Efficacy Scale (GSE‐17). Participants were over 18 years old, Persian‐speaking, willing to participate, had no hearing or speech impairments, and completed at least 90% of the questionnaires. Statistical analysis was performed using SPSS 24, including descriptive statistics, correlation test, t‐test, ANOVA, and stepwise multiple linear regression to identify predictors of anxiety and self‐efficacy.

**Results:**

The mean age of the 120 caregivers was 40.15 ± 11.75 years, with most being female (74.2%). The mean anxiety score was 21.96 ± 8.13, while the mean self‐efficacy score was 55.98 ± 6.38. Anxiety and self‐efficacy showed a significant negative correlation (r = –0.449, 95% CI –0.58 to –0.29, *p* < 0.001). Regression analysis revealed that caregiver education, age, sex, self‐efficacy, and patient residence predicted anxiety (adjusted R² = 42.1%, *p* < 0.001). In contrast, caregiver age, anxiety, and patient marital status, addiction history, and ostomy type predicted self‐efficacy (adjusted R² = 28.2%, *p* < 0.001).

**Conclusions:**

Our study showed a correlation between anxiety and self‐efficacy among caregivers of patients with ostomy. We also found that certain sociodemographic factors predict levels of anxiety and self‐efficacy. Therefore, healthcare workers should assess caregivers' anxiety and self‐efficacy levels, foster a positive environment, and improve overall quality of life for both caregivers and patients.

## 1 Introduction

Intestinal stomas, artificial openings in the bowel, can be either temporary or permanent [1]. Approximately 30% of patients with advanced‐stage colorectal cancer undergo ostomy surgery to divert feces away from affected organs [2]. Many of these patients have limited self‐management skills [3], which often lead to family members assuming a primary caregiving role. These caregivers are responsible for a wide range of tasks, including symptom management, physical care, and medication administration [4]. This significant burden can impact the quality of life for both caregivers and patients [5]. Thus, preparing, involving, and supporting caregivers is crucial for enhancing patient quality of life and overall recovery [3].

Stoma surgery can have a significant impact on the physical and mental health of patients and caregivers [3]. The stress associated with ostomy can impair coping abilities and lead to increased anxiety in caregivers [6]. Anxiety, characterized by fear, dread, and excessive worry, can manifest as emotional, behavioral, and cognitive symptoms [7]. Previous studies have shown higher rates of anxiety symptoms in patients with ostomy and their caregivers [3, 8, 9, 10, 11].

To adapt to the physical and psychological changes of living with ostomy, both patients and caregivers need adequate self‐efficacy [12]. Self‐efficacy is the belief in one's ability to take action and accomplish goals, which empowers individuals to overcome obstacles and adopt healthy behaviors such as self‐care skills [13]. Stoma self‐efficacy reflects the confidence patients and caregivers have in managing their stomas to minimize negative outcomes [14]. Research shows that self‐efficacy is a key factor in successful stoma self‐management and a strong predictor of positive care outcomes [15].

Various studies have examined the specific factors that affect anxiety and self‐efficacy in individuals with stomas. For example, self‐efficacy is positively influenced by factors such as self‐esteem [16], a higher education level, reduced economic burdens, and increased social support [17]. Other contributing factors are the duration of having a stoma [18], emotional intelligence, being married [19], and experiencing a positive psychosocial impact [20]. Additionally, self‐efficacy [18] and participation in rehabilitation programs [21] have been shown to have a beneficial effect on anxiety levels.

The challenges of living with an ostomy extend beyond the physical management of the stoma. Individuals often experience significant lifestyle disruptions, including a decline in physical activity due to concerns about stoma‐related complications such as hernia, appliance leakage, or discomfort, which in turn negatively affect psychological well‐being and quality of life [22]. Sexual functioning and intimacy are also commonly impaired following stoma formation. Studies report high rates of sexual dysfunction (e.g., up to 90% on ASEX scales) and lower satisfaction, both of which correlate with diminished self‐efficacy in adapting to stoma‐related changes [23]. Furthermore, alterations in body image and confidence, including feelings of stigma or self‐esteem decline, may exacerbate anxiety and undermine self‐efficacy, highlighting the need to consider physical, intimate, and psychosocial dimensions when evaluating mental health and coping capabilities of both caregivers and patients [24]

Research on anxiety, self‐efficacy, and their predictors among caregivers of patients with ostomy is limited. William Goodman et al. examined how self‐efficacy and quality of life in individuals with stomas fluctuate throughout the day. These variations are influenced by contextual factors such as location and time. This was the first study to utilize ecological momentary assessment (EMA) in this particular population [25]. The study conducted by Juan‐Ying Ding et al. demonstrated that peer‐led interventions significantly enhanced patients' knowledge, attitudes, and practices related to ostomy care, leading to better quality of life and fewer complications. These findings suggest that such interventions are an effective approach for individuals living with permanent ostomies [26]. While direct research on caregivers of ostomy patients is scarce, relevant findings can be drawn from studies on caregivers of cancer patients. For example, a systematic review by Thomas Hebdon et al. and a survey by Mystakidou et al. investigated the relationship between caregivers' anxiety and self‐efficacy [27, 28]. A systematic review by Hebdon et al. and a survey by Mystakidou et al. explored the association between caregivers' anxiety and self‐efficacy [24, 25]. Additionally, a survey by Karabekiroğlu et al. and a study by Karimi Moghaddam et al. reported high levels of anxiety among caregivers of cancer patients [29, 30].

A body of research by Liu et al., Kizza et al., Havyer et al., Leung et al., and Phongtankuel et al. has shown that higher caregiver self‐efficacy is associated with improved caregiving roles, better patient outcomes, and enhanced caregiver well‐being. These studies also identified significant predictors of self‐efficacy, including the caregiver's health status, knowledge, caregiving demands, and the patient's functional status [5, 31, 32, 33, 34]. This evidence underscores the importance of integrating strategies that boost caregiver self‐efficacy into intervention programs.

Ostomy surgery can significantly impact the physical and psychological well‐being of both patients and their caregivers. Given that caregivers play a crucial role in supporting patients who cannot manage their own care, the quality of care they provide directly impacts the patient's quality of life. To improve patient outcomes, healthcare professionals must address caregivers' psychological well‐being such as anxiety. This study aimed to assess anxiety, self‐efficacy, and their predictors among caregivers of patients with ostomy.

## 2 Methods and Materials

### 2.1 Study Design and Setting

This descriptive‐correlational cross‐sectional study was conducted on primary caregivers of patients with stomas visiting the stoma clinic in Kerman province, Iran, between May 2, 2023, and July 20, 2024.

### 2.2 Sample Size and Sampling

The sample size was estimated based on a 95% confidence level, 80% power, and a medium effect size (r = 0.25) for correlation analysis, using G*Power software. According to this estimation, a minimum of 123 participants was required. To account for potential dropout or incomplete responses, 131 participants were recruited using convenience sampling.

**Figure 1:**
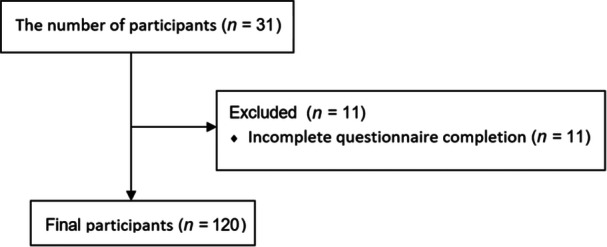
Flow Diagram.

Inclusion criteria were: age 18 years or older, ability to speak Persian, willingness to participate, and no hearing or speech impairments. Participants who did not complete at least 90% of the questionnaires were excluded from the analysis. Out of 131 participants in this study, 11 questionnaires were excluded due to incomplete completion, and finally, 120 questionnaires were analyzed (Figure 1).

### 2.3 Measures

In the present study, the following questionnaires were utilized:

**Demographic Information Questionnaire**: This tool collected detailed background information from both caregivers and patients. For caregivers, data included age, sex, marital status, education level, occupation, monthly income, place of residence, medical history, smoking status, and duration of caregiving. For patients, data included age, sex, type and location of ostomy, ostomy duration, marital status, history of addiction or chronic illness, body mass index (BMI), and current medications.

**Hamilton Anxiety Rating Scale (HAM‐A):** The HAM‐A is a 14‐item clinician‐rated scale that assesses the severity of psychological and somatic anxiety symptoms. Each item is scored on a scale from 0 (not present) to 4 (very severe), yielding a total score ranging from 0 to 56. Scores below 17 indicate mild anxiety, 18–24 mild to moderate anxiety, 25–30 moderate to severe anxiety, and scores above 30 indicate severe anxiety. The Persian version of the HAM‐A has demonstrated good internal consistency (Cronbach's alpha = 0.88) and acceptable content validity in Iranian populations [35].

**Generalized Self‐Efficacy Scale (GSES):** We used the 17‐item GSES, designed by Sherer, to measure self‐efficacy. Scores range from 17 to 85, with higher scores indicating greater self‐efficacy. Items are scored using a five‐point Likert scale (from strongly disagree to strongly agree). Items 1, 3, 8, 9, 13, and 15 are scored directly, with higher scores indicating greater self‐efficacy. The remaining questions are scored in reverse, with lower scores indicating greater self‐efficacy. The original English version has a Cronbach's alpha of 0.86 [36]. The Persian versions of both the HAM‐A and GSES have been validated in previous Iranian studies, demonstrating excellent psychometric properties, including strong internal consistency and validity [37, 38].

The internal consistency of the Hamilton Anxiety Rating Scale (HAM‐A) in our sample was Cronbach's α = 0.804, and for the Generalized Self‐Efficacy Scale (GSES‐17) was Cronbach's α = 0.789.

### 2.4 Data Collection

Primary caregivers of patients with stomas visiting the stoma clinic in Kerman province were selected according to the inclusion criteria. The demographic questionnaire was completed by the participants in this research. To prevent any bias in this study, the Hamilton Anxiety Rating Scale (HAM‐A) and the Generalized Self‐Efficacy Scale (GSES) were completed by a trained individual through interviews with the participants. The researcher provided contact information for any questions or concerns. Sampling took place from May 2, 2023 to July 20, 2024.

### 2.5 Statistical Analysis

Data were analyzed using SPSS version 24. Descriptive statistics, including mean, standard deviation, frequency, percentage, and range, were used to describe participants' characteristics and main outcome variables (anxiety and self‐efficacy). Pearson's correlation coefficient was applied to assess relationships between continuous variables. Independent t‐test and one‐way ANOVA were used to compare anxiety and self‐efficacy scores across categorical groups (e.g., gender, marital status). Given the lack of prior research specifically on correlates of anxiety (HAM‐A) and self‑efficacy (GSES) in ostomy caregivers, an exploratory approach using stepwise multiple linear regression was adopted. The aim was to generate hypotheses rather than to test preconceived causal models. A *p*‐value of < 0.05 was considered statistically significant in all analyses. Statistical analyses, reporting, and interpretation in this study were conducted in accordance with the recommendations outlined in the “Guidelines for Reporting of Statistics for Clinical Research in Urology” by Assel et al. (2018) to ensure methodological rigor and clarity [39]. Range was used to report the minimum and maximum values for continuous variables.

### 2.6 Ethical Considerations

This study adhered to the Declaration of Helsinki. Ethical approval was granted by the Ethics Committee of Kerman University of Medical Sciences (IR.KMU.REC.1401.379). Written informed consent was obtained from all participants, and confidentiality was ensured throughout the study. Participants were informed of their right to withdraw at any time, and overall findings were shared with those interested. All methods were carried out in accordance with relevant guidelines and regulations.

## 3 Results

### 3.1 Sociodemographic Characteristics

#### 3.1.1 Caregivers

A total of 120 caregivers participated in the study. The mean age was 40.15 ± 11.75 years (range: 18–67), and the mean duration of caregiving was 26.65 ± 13.69 months. The majority were female (*n* = 89, 74.2%), married (*n* = 71, 59.2%), and had a diploma or higher education level (*n* = 97, 80.8%). Regarding occupation, 58 caregivers (48.3%) were unemployed, 56 (46.7%) were employed, and 6 (5.0%) were retired. Most of the caregivers lived in Kerman province (*n* = 61, 50.8%) and reported no history of chronic disease (*n* = 99, 82.5%). Monthly income distribution was as follows: ≤ 3 million Toman (*n* = 54, 45.0%), 3–6 million (*n* = 10, 8.3%), and > 6 million (*n* = 56, 46.7%) (Table 1).

**Table 1:** The association between anxiety and self‐efficacy in caregivers.

Variables	Mean (SD)/*n* (%)	Anxiety [statistical test (*p* value)]	Self‐efficacy [statistical test (*p* value)]
Age (yr.)	40.15 (11.75)	r = 0.418 ( < 0.001)	r = −0.308 (0.001)
Duration of caregiving (month)	26.65 (13.69)	r = 0.188 (0.040)	r = −0.018 (0.849)
**Caregiver sex**			
Male	31 (25.8)	t = −2.902 (0.004)	t = 1.538 (0.127)
Female	89 (74.2)		
**Marital status**			
Married	71 (59.2)		
Single	27 (22.5)	F = 10.472 (< 0.001)	F = 5.718 (0.004)
Other	22 (18.3)		
**Education**			
Illiterate	5 (4.2)		
Lower secondary	18 (15.0)	F = 15.673 (< 0.001)	F = 4.017 (0.009)
Diploma	33 (27.5)		
Above diploma	64 (53.3)		
**Job**			
Unemployed	58 (48.3)		
Employed	56 (46.7)	F = 9.866 (< 0.001)	F = 3.780 (0.026)
Retired	6 (5.0)		
**Living place**			
Kerman	61 (50.8)	t = −1.112 (0.268)	t = −0.270 (0.787)
Others	59 (49.2)		
**Income (million toman)**			
≤ 3	54 (45.0)		
3 − 6	10 (8.3)	F = 10.106 (< 0.001)	F = 4.275 (0.016)
> 6	56 (46.7)		
**History of chronic disease**			
No	99 (82.5)	t = −3.871 (< 0.001)	t = 2.320 (0.022)
Yes	21 (17.5)		

*Note:* r = Pearson correlation coefficient, t = Independent t test, F = Analysis of variance SD: standard deviation for continuous variables. *n* (%): Values for categorical variables were presented as frequencies (percentages). *p*‐value: *p*‐values less than 0.05 were considered statistically significant.

### 3.2 Patients

The mean age of patients was 53.89 ± 18.96 years. Most of them were male (*n* = 72, 60.0%), married (*n* = 90, 75.0%), and unemployed (*n* = 69, 57.5%). The majority had a permanent ostomy (*n* = 105, 87.5%) and a colostomy (*n* = 96, 80.0%), with an average ostomy duration of 27.53 ± 13.46 months. Regarding health status, 61 patients (50.8%) reported chronic diseases, and 35 (29.2%) had a history of addiction (Table 2).

**Table 2:** The patients' characteristics and caregivers' anxiety and self‐efficacy.

Variables	Mean (SD)/*n* (%)	Anxiety [statistical test (*p* value)]	Self‐efficacy [statistical test (*p* value)]
Age (yr.)	53.89 (18.96)	r = −0.208 (0.023)	r = 0.009 (0.919)
Body mass index	24.70 (3.17)	r = −0.154 (0.093)	r = 0.230 (0.012)
Duration of having ostomy (months)	27.53 (13.46)	r = 0.261 (0.004)	r = −0.139 (0.131)
**Sex**			
Male	72 (60.0)	t = 1.102 (0.273)	t = −0.706 (0.482)
Female	48 (40.0)		
**Marital status**			
Married	90 (75.0)		
Single	14 (11.7)	F = 4.652 (0.011)	F = 1.592 (0.208)
Other	16 (13.3)		
**Education**			
Illiterate	23 (19.2)		
Lower secondary	35 (29.2)	F = 0.375 (0.771)	F = 0.379 (0.769)
Diploma	26 (21.6)		
Higher levels	36 (30.0)		
**Job**			
Unemployed	69 (57.5)		
Employed	22 (18.3)	F = 0.049 (0.952)	F = 1.055 (0.351)
Retired	29 (24.2)		
**Living place**			
Kerman	60 (50.0)	t = −1.250 (0.214)	t = −0.214 (0.831)
Others	60 (50.0)		
**Income (million toman)**			
≤ 3	58 (48.3)		
3–6	23 (19.2)	F = 0.701 (0.498)	F = 0.242 (0.785)
> 6	39 (32.5)		
**History of addiction**			
No	85 (70.8)	t = −0.504 (0.615)	t = 1.980 (0.050)
Yes	35 (29.2)		
**History of chronic disease**			
No	59 (49.2)	t = 2.084 (0.039)	t = −0.557 (0.578)
Yes	61 (50.8)		
**Type of ostomy**			
Permanent	105 (87.5)	t = −1.946 (0.054)	t = 1.506 (0.135)
Temporary	15 (12.5)		
**Ostomy location**			
Colostomy	96 (80.0)	t = 1.410 (0.161)	t = −2.317 (0.022)
Ileostomy	24 (20.0)		

*Note:* r = Pearson correlation coefficient, t = Independent t test, F = Analysis of variance.

### 3.3 Descriptive Findings of Main Outcomes

The mean anxiety score among caregivers was 21.96 ± 8.13 (range: 1–47), which was below the midpoint of the HAM‐A scale [28]. The mean self‐efficacy score was 55.98 ± 6.38 (range: 37–71). Based on GSES cut‐offs, 24.2% of caregivers had moderate self‐efficacy and 75.8% had high self‐efficacy (Table 1).

A statistically significant negative correlation was found between anxiety and self‐efficacy (r = –0.449, *p* < 0.001), indicating that higher anxiety levels were associated with lower self‐efficacy (Table 1).

## 4 Inferential Findings

### 4.1 Anxiety

Bivariate analyses showed that anxiety was significantly associated with caregiver age, duration of care, sex, marital status, education, job status, monthly income, and history of chronic disease (Table 1). Patient‐related factors also played a role, with patient age, marital status, and history of chronic disease showing a significant association with caregiver anxiety (Table 2).

Stepwise multiple regression analysis identified caregiver education level, age, sex, self‐efficacy, and patient place of residence as significant predictors of caregiver anxiety (adjusted R² = 42.1%, *p* < 0.05). Higher caregiver age and female sex predicted higher anxiety, while higher self‐efficacy predicted lower anxiety. Caregivers of patients living outside Kerman reported higher anxiety levels (Table 3).

**Table 3:** The predictors of the anxiety score of caregivers of patients with ostomy.

Variable		B	SE^a^	β	T	*p*	95% CI Lower	95% CI Upper	Adjusted R^2^
Anxiety	Content	25.915	7.326		3.537	0.001	11.401	40.428	0.421*
	Caregiver education level (Above diploma vs. Illiterate)	−2.410	1.365	−0.149	−1.766	0.080	−5.113	0.293	
	Caregiver education level (Under diploma vs. Illiterate)	5.758	1.80	0.254	3.199	0.002	2.192	9.324	
	Caregiver age	0.148	0.054	0.214	2.723	0.007	0.040	0.255	
	Caregiver sex (Male = 1, female = 2)	3.082	1.329	0.167	2.319	0.022	0.449	5.716	
	Caregiver self‐efficacy	−0.332	0.096	−0.261	−3.462	0.001	−0.523	−0.142	
	Patient living place (Kerman = 1, Others = 2)	2.514	1.141	0.155	2.203	0.030	0.253	4.775	

* F = 15.416, *p* < 0.001.

^a^ Standard error. CI, Confidence intervals for B.

### 4.2 Self‐Efficacy

Bivariate analyses showed that self‐efficacy was significantly associated with caregiver age, marital status, education, job status, monthly income, and history of chronic disease (Table 1). Patient‐related factors also played a role, with BMI and ostomy location showing a significant correlation with caregiver self‐efficacy (Table 2).

Stepwise multiple regression analysis identified caregiver age, anxiety score, patient marital status, history of addiction, and ostomy location as significant predictors of caregiver self‐efficacy (adjusted R² = 28.2%, *p* < 0.05). Higher caregiver age and anxiety predicted lower self‐efficacy. Caregivers of unmarried patients and those with a history of addiction reported lower self‐efficacy, while caregivers of patients with ileostomy reported higher self‐efficacy than those with colostomy (Table 4).

**Table 4:** The predictors of the self‐efficacy score in caregivers of patients with ostomy.

Variable		B	SE^a^	β	T	P	95% CI Lower	95% CI Upper	Adjusted R^2^
**Self‐efficacy**	Constant	63.345	2.483		25.508	< 0.001	58.426	68.265	0.282*
	Caregiver age	−0.108	0.047	−0.199	−2.277	0.025	−0.202	−0.014	
	Patient marital status (other vs. married)	−3.189	1.480	−0.171	−2.155	0.033	−6.121	−0.257	
	Patient history of addiction (No = 0, Yes = 1)	−2.519	1.111	−0.180	−2.268	0.025	−4.720	−0.318	
	Ostomy location (Colostomy = 1, Ileostomy = 2)	3.271	1.275	0.206	2.565	0.012	0.745	5.797	
	Anxiety score	−0.264	0.068	−0.336	−3.868	< 0.001	−0.399	−0.129	

* F = 10.333, *p* < 0.001.

^a^ Standard error. CI, Confidence intervals for B.

## 5 Discussion

Recent study results demonstrate a moderate yet significant association between anxiety and self‐efficacy. In this sample, higher anxiety scores were accompanied by lower self‐efficacy, which is consistent with previous research underscoring the relevance of self‐efficacy in coping with anxiety [28, 40, 41, 42, 43, 44]. For example, Özden and Kılıç reported that higher self‐efficacy was associated with better stoma adaptation [41]. In addition, Gong et al. confirmed a negative correlation between anxiety and self‐efficacy [42]. Findings from research on chronic conditions similarly suggest that self‐efficacy is an important component of self‐management and may relate to more effective stoma‐related healthcare management. Strengthening self‐management‐related skills for both patients with a stoma and their caregivers may support smoother recovery and psychosocial adaptation to a new lifestyle. Self‐management shifts the focus from complete reliance on the healthcare system toward enabling patients and caregivers to oversee their own condition. Such an approach is expected to support autonomy by fostering the skills required for long‐term management [17]. Accordingly, interventions targeting self‐efficacy may be beneficial in supporting anxiety‐related outcomes among ostomy patients and their caregivers [43].

The present results also indicated that caregiver education level, age, sex, self‐efficacy, and patient living place were associated with caregivers' anxiety level. Giordano et al. highlighted that caregivers with lower educational levels were less effective in patient care and tended to report lower self‐efficacy [45]. Although we anticipated a negative association between self‐efficacy and anxiety, our findings showed an unexpected pattern: caregivers with lower secondary education reported significantly lower anxiety scores compared with those who were illiterate. Several studies have reported associations between higher caregiver anxiety and lower educational backgrounds [44, 46, 47, 48, 49, 50]. One possible interpretation is that caregivers with higher educational levels may be more informed about disease prognosis and related challenges, which could be associated with heightened anxiety. Conversely, caregivers with lower educational levels—particularly those who are illiterate or have only elementary education—may experience greater barriers to engaging with diagnostic and treatment processes. This may impede effective communication with healthcare providers and insurance systems [30]. Understanding how education may relate to caregiver anxiety can help practitioners identify and address anxiety‐related concerns more effectively [46].

This study is consistent with prior evidence suggesting that female caregivers experience higher levels of anxiety than male caregivers. It also underscores how a family member's illness can influence relationships and caregiving roles, particularly for women who often play a central caregiving role. A previous study reported that the caregiving burden among family caregivers of patients with a stoma was generally mild to moderate. It further observed that increasing caregiving burden was associated with higher levels of anxiety and depression among caregivers [28, 30, 44, 47, 49, 50, 51, 52, 53]. Hu et al. reported similar findings and emphasized that healthcare providers may need to focus on women and implement tailored approaches—such as psychological counseling and technical training/education—to alleviate anxiety‐related difficulties [49].

Caregivers of patients living outside Kerman reported higher anxiety levels, which aligns with a previous study [47]. Addressing this pattern may require the development of locally based, community‐oriented mental health strategies aimed at promoting mental wellbeing and preventing mental illness [47]. In addition, providing caregivers with relevant information and resources may help them overcome logistical barriers (e.g., time constraints and transportation issues) when seeking access to behavioral health services [54].

Our study also revealed a significant positive association between age and anxiety levels, consistent with findings from other studies [44, 50, 55, 56]. Pucciarelli et al. similarly reported that older age was related to higher anxiety [55]. The observed prevalence of psychological consequences among family caregivers may reflect the challenges they face and the difficult realities they must accept. One possible explanation is that caregivers may be reluctant to fully express the extent of their burdens due to their close relationship with the patient. Moreover, we observed a significant negative association between age and self‐efficacy, which is consistent with previous research [44]. Greene et al. emphasized the substantial time and effort young adults may invest in caring for dependent family members [54], which might relate to higher self‐efficacy levels.

Caregivers of unmarried patients reported lower self‐efficacy compared with caregivers of married patients, consistent with earlier findings [44, 56, 57]. Spousal support may remain an important aspect of family caregiving [58]. While extended family and friends can provide social support, opportunities for shared problem‐solving, and opportunities to learn [47], caregivers who perceived significant family dysfunctions reported higher anxiety and lower self‐efficacy [28, 31, 57, 59].

Caregivers of patients with a history of addiction reported lower self‐efficacy compared with caregivers of patients without such a history. Research has indicated relationships among abstinence motivation, self‐efficacy, and drug addiction [60, 61, 62, 63, 64]. Increasing self‐control, resilience, and self‐esteem may support self‐efficacy among individuals with substance use disorders [65]. In addition, family‐centered empowerment interventions have been shown to improve caregivers' health literacy, self‐efficacy, and overall quality of care [66].

In this study, caregivers of patients with ileostomies reported higher self‐efficacy than caregivers of patients with colostomies. However, this finding contrasts with a study by Simmons et al., which reported that participants with rectal stomas faced greater adjustment challenges [67]. This suggests the importance of individualized care based on specific disease conditions. Prior research has also emphasized that caregivers may need sufficient disease‐related information to make informed care decisions [68]. Further research is necessary to clarify the observed discrepancy in self‐efficacy levels between ileostomy and colostomy caregivers.

This study had several limitations that warrant consideration when interpreting the findings. First, the cross‐sectional design limited our ability to determine causality between risk factors, self‐efficacy, and anxiety, as it only captures a snapshot in time. Second, due to the relatively small number of patients with a stoma, we were unable to obtain a large sample size, which may impact the statistical power to detect smaller effects or conduct more complex subgroup analyses. Third, the use of stepwise regression in this study was exploratory. Stepwise procedures are known to produce unstable estimates, overfit small samples, and inflate the risk of false positives. Therefore, our findings should be interpreted as hypothesis‑generating rather than confirmatory. Replication in larger, independent samples is needed to validate the identified correlates. Fourth, our reliance on self‐reported responses, while common in such research, may have introduced response bias, potentially influencing the accuracy of the data collected. Additionally, the study may have omitted some relevant variables that could affect the relationships under investigation, potentially biasing the results. Finally, our study utilized a convenience sample from Iran, which, given the specific cultural and socio‐economic context, may limit the generalizability of the findings to the broader international ostomy population. Future research employing longitudinal designs, larger and more diverse samples, and potentially objective measures could address these limitations.

## 6 Conclusion

Our study indicates a correlation between anxiety and self‐efficacy among caregivers of patients with ostomies. Additionally, we identified specific sociodemographic factors that influence these levels. To enhance the well‐being of both caregivers and patients, nurses should prioritize assessing caregiver anxiety and self‐efficacy, fostering a supportive environment, and striving to improve overall quality of life.

## Author Contributions

**Sima Mokhtarabadi:** conceptualization, data curation, investigation, methodology, writing – review and editing. **Ala Shamsi:** visualization, writing – original draft, investigation. **Haadiyeh Rahbari:** data curation, visualization, methodology, writing – review and editing. **Marzie Mosa Ali:** data curation, visualization, methodology, writing – review and editing. **Mohamed Alnaiem:** writing – review and editing, validation, methodology. **Mahlagha Dehghan:** conceptualization, investigation, methodology, supervision, writing – review and editing, data curation, formal analysis.

## Funding

The authors have nothing to report.

## Conflicts of Interest

The authors declare no conflicts of interest.

## Transparency Statement

The lead author Mahlagha Dehghan affirms that this manuscript is an honest, accurate, and transparent account of the study being reported; that no important aspects of the study have been omitted; and that any discrepancies from the study as planned (and, if relevant, registered) have been explained.

## Data Availability

The data that support the findings of this study are available from the corresponding author upon reasonable request.
